# Salcaprozate Sodium as a Permeation Enhancer—Mechanism, Applications and Unresolved Questions

**DOI:** 10.3390/pharmaceutics18091171

**Published:** 2026-09-16

**Authors:** Boris Dinkov, Nikolinka Koleva, Galya Stavreva

**Affiliations:** 1 Department of Pharmacology and Toxicology, Faculty of Pharmacy, Medical University, 5800 Pleven, Bulgaria; 2 Clinic of Endocrinology and Metabolic Diseases, University Hospital “Dr. Georgi Stranski”, 5800 Pleven, Bulgaria; 3 Center of Competence in Personalized Medicine, 3D and Telemedicine, Robotic and Minimally Invasive Surgery “Leonardo da Vinci”, Medical University, 5800 Pleven, Bulgaria

**Keywords:** salcaprozate sodium, SNAC, permeation enhancer, oral peptide delivery, oral bioavailability, gastric absorption

## Abstract

Oral absorption remains challenging for peptides, proteins, and other drug substances with limited stability or permeability. Sodium N-(8-[2-hydroxybenzoyl]amino)caprylate (SNAC), also known as salcaprozate sodium, is an oral permeation enhancer used in approved drug products. This review summarizes the physicochemical properties and mechanisms of action of SNAC and evaluates its clinical and emerging preclinical applications. A structured literature search was conducted in PubMed and Scopus, supplemented as needed with ClinicalTrials.gov and regulatory documents. The search covered 2015–2026, with earlier key publications included where necessary for historical context. After screening, 71 sources were included in the narrative synthesis. The review examines the proposed mechanisms of SNAC-mediated permeation enhancement, including local pH modulation, membrane-mediated effects, peptide self-association, and context-dependent effects on epithelial permeability. Clinical evidence is summarized for oral semaglutide, cyanocobalamin, unfractionated heparin, ibandronate, and investigational peptide products. Emerging applications beyond metabolic diseases are discussed together with formulation strategies and safety considerations. Recent preclinical findings suggesting changes in gut microbiota and inflammatory markers are critically evaluated in the context of the available clinical safety experience, while their relevance to humans remains unconfirmed. Overall, SNAC reliably enhances permeation in vitro and in vivo, yet the resulting oral bioavailability remains low and variable and is strongly dependent on payload, formulation, and gastrointestinal conditions. Long-term safety and the clinical relevance of recent preclinical microbiome findings remain important areas for future investigation.

## 1. Introduction

The oral route of drug administration is preferred for its convenience, non-invasiveness, and improved patient adherence in the treatment of chronic diseases [1]. For certain drug substances, such as peptides, proteins, and unstable drugs, absorption is significantly hindered by multiple factors within the gastrointestinal (GI) tract. These factors can lead to denaturation due to low pH, enzymatic degradation, and reduced epithelial permeability [1].

The oral bioavailability of peptide- and protein-based drugs is limited, primarily by presystemic degradation [2,3]. The acidic environment of the stomach and the action of proteolytic enzymes (pepsin, trypsin, chymotrypsin and peptidases) rapidly degrade the peptide chain. Second, the permeability across the GI mucosa is reduced. The large molecular mass, hydrophilicity, and charge of the drug molecule can hinder its passage via both transcellular and paracellular routes. As a result, the oral bioavailability of most native peptides in adults is negligible [4].

Strategies for improving oral bioavailability include the use of enzyme inhibitors, mucoadhesive systems, nanoparticles, and enteric coatings [5]. Among these approaches, particular attention has been given to permeation enhancers (PEs) [6], which can temporarily and reversibly increase gastrointestinal epithelial permeability to drug substances. Sodium N-(8-[2-hydroxybenzoyl]amino)caprylate (SNAC) is an excipient developed within the “delivery agent approach” [2,7], in which a carrier molecule interacts noncovalently with the active substance. SNAC has subsequently been developed as an oral permeation enhancer and incorporated into several drug products [2,7].

SNAC originated from Emisphere Technologies’ research program on synthetic “delivery agents” [8]. In 2009, SNAC received GRAS status (generally recognized as safe) for use with nutritional substances, with a safe daily intake of up to 250 mg in a nutritional context [9]. Eligen B12 (cyanocobalamin formulated with SNAC) was the first marketed product using SNAC-based carrier technology. It was marketed in the United States as a medical food for use under medical supervision, preceding the subsequent development of SNAC as a permeation enhancer for oral semaglutide [2].

**Figure 1:**
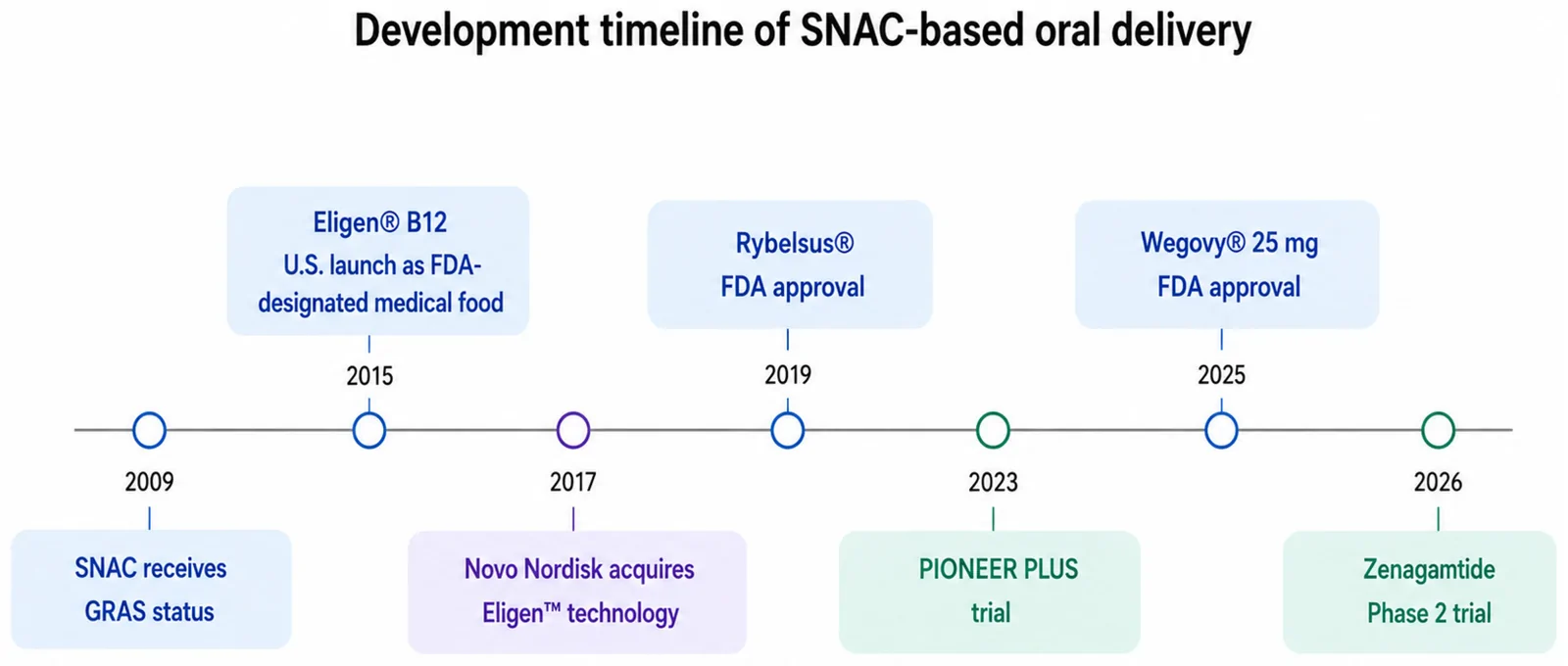
Development timeline of the SNAC-based oral delivery platform (2009–2026), highlighting regulatory, platform-development, and clinical-development milestones. Abbreviations: FDA—U.S. Food and Drug Administration; GRAS—generally recognized as safe.

In 2017, Novo Nordisk acquired the Eligen™ technology from Emisphere and applied it to the development of oral GLP-1 receptor agonists (GLP-1RAs) [10]. A major milestone was reached in 2019 with the approval of once-daily oral semaglutide (Rybelsus^®^) [10,11], the first oral peptide therapy of the GLP-1RA class based on SNAC technology. This represented an important milestone for both the treatment of T2DM and the development of oral peptide delivery. SNAC has since been investigated in a broader range of applications, including new peptide and non-peptide payloads and alternative dosage forms such as mucoadhesive tablets and liquid formulations (Figure 1).

Although oral semaglutide remains the most clinically established application of SNAC, the present review considers the platform in a broader context, encompassing its physicochemical properties, mechanisms of action, clinical applications, emerging uses, and unresolved safety questions. This broader perspective reflects the continuing development of SNAC as an oral permeation-enhancing platform.

## 2. Methods

A structured literature search was conducted in the PubMed and Scopus databases, supplemented as needed with ClinicalTrials.gov for information on ongoing and completed clinical trials. Since SNAC has gained considerable relevance in recent years, primarily in connection with the expanding clinical use of oral semaglutide, the search covered the period from 1 January 2015 to 31 July 2026, with the exception of historically key publications from earlier years necessary to provide context for the development of the technology. Keywords included “salcaprozate sodium,” “SNAC,” “permeation enhancer,” “absorption enhancer,” “oral bioavailability,” “semaglutide,” and “oral peptide delivery”. The search scope included original studies (in vitro, ex vivo, in vivo, preclinical, and clinical studies), literature reviews, and regulatory documents (FDA/EMA) published in English. Publications without direct relevance to SNAC or its mechanism of action, conference abstracts without full text, and duplicate records across databases were excluded. A total of 82 records were identified. After screening titles and abstracts, 11 records were excluded as duplicates or not directly relevant to the scope of the review, yielding 71 included sources for the narrative synthesis. Due to the considerable heterogeneity of the available evidence, including differences in drug payloads, experimental models, dosages, and study methodologies, quantitative synthesis was not considered appropriate, and a narrative review approach was therefore adopted.

## 3. Chemical Structure and Physicochemical Properties

SNAC (CAS RN 203787-91-1; sodium 8-((2-hydroxybenzoyl)amino)octanoate; molecular formula C_15_H_20_NNaO_4_; molecular mass 301.31 g/mol) is a synthetic N-acylated derivative of octanoic acid [6]. The structure of SNAC incorporates salicylic (2-hydroxybenzoic) acid, amide-linked to an eight-carbon (caprylic/octanoic) fatty acid chain, whose carboxyl group is present as a sodium salt [2]. The molecule combines a hydrophobic aliphatic segment with a polar salicylic “head.” This structure confers amphiphilic properties and activity characteristic of surface-active agents [2].

SNAC is the sodium salt of a weak acid (pKa ≈ 5.01) [6,12]. It is a white crystalline powder, with solubility strongly dependent on the pH of the medium [2,6,12]. This pH dependence has direct functional significance: since effective membrane fluidization requires the PE to be in monomeric form, the critical micelle concentration (CMC) is a key determinant of efficacy [13]. SNAC does not form micelles in the stomach due to protonation, but forms micelles at intestinal pH, with electrolytes further lowering the critical micelle concentration. This dynamic is important for maintaining the monomeric, membrane-active form [13]. Bile salts exhibit a biphasic effect—raising the CMC at low concentrations and promoting mixed micellization at higher concentrations [13].

The amphiphilic character underlies both the molecule’s ability to associate with protein molecules and its interaction with the lipid membranes of GI epithelial cells. These physicochemical characteristics are directly reflected in the molecule’s specific biophysical interactions.

### 3.1. Amphiphilic Character

SNAC has been shown to behave primarily as a surfactant. This has been demonstrated in head-to-head comparisons with Caco-2 (a cell line that spontaneously forms a monolayer structurally and functionally resembling the intestinal epithelium) against sodium caprate (C10). Both agents exhibit detergent-like properties with initial membrane fluidization [14]. C10 reversibly decreases transepithelial electrical resistance (TEER), induces reorganization of three tight-junction proteins, and increases permeability to FITC-dextran 4000 (a marker of paracellular permeability). In contrast to C10, SNAC does not increase paracellular flux in Caco-2 monolayers at non-cytotoxic concentrations [14]. SNAC has been found to affect only the localization of claudin-5 [14].

Data from isolated tissues, however, are not fully consistent with cell-based models. In isolated rat gastric mucosa, SNAC increases the apparent permeability coefficient (Papp) of FD4 in a concentration-dependent manner. This effect is not observed with C10 in the gastric mucosa. In the jejunum and colon, both SNAC and C10 increase the Papp of both mannitol and FITC-dextran 4000, although achieving the efficacy of 10 mM C10 requires 20 mM SNAC [14,15].

Taken together, these data support a predominantly transcellular/membrane-mediated mechanism of action in cell monolayers, alongside a measurable paracellular component in isolated mucosae, particularly depending on the anatomical region of the GI tract [14,15].

### 3.2. Interaction with Lipid Membranes

Solid-state NMR (ssNMR) data in a hydrated DMPC liposome model show that SNAC increases the uniaxial mobility of phospholipids in the membrane layer in a concentration-dependent manner. Headgroup dynamics reach a plateau of approximately 25% between 24 and 72 mM. At the same time, it produces a 43% increase in the fluidity of the hydrophobic core at 72 mM [16]. The effect of SNAC is therefore considerably stronger deep within the membrane (the hydrophobic core) than near the surface.

Two limitations of the model should be noted. The degree of liposome dissolution and conversion into micelles increases with SNAC concentration. In the presence of the model peptide octreotide, the increase in lipid mobility is weaker and lacks the expected plateau effect. This is explained by a reduction in monomeric SNAC due to SNAC-peptide interactions, giving rise to a competitive equilibrium within the SNAC-lipid–peptide system [16].

Molecular dynamics simulations provide a complementary but fundamentally different measure of membrane dynamics from ssNMR. Whereas ssNMR measures uniaxial mobility of lipid segments, MD simulations quantify lateral diffusion within the membrane plane; these parameters should not be treated as interchangeable measures of “fluidity.” In the ssNMR experiments, SNAC increases uniaxial lipid mobility in a concentration-dependent manner [16]. By contrast, MD simulations show that SNAC decreases lateral lipid diffusion, whereas C10 increases it—yet SNAC nonetheless lowers the central free-energy barrier to transmembrane permeation more effectively than C10 [17,18]. Taken together, an increase in uniaxial mobility does not necessarily correspond to an increase in lateral membrane fluidity, and the term “fluidization” should therefore be used with the experimental modality specified, rather than as a general descriptor of all changes in membrane dynamics.

Molecular dynamics simulations have also suggested a defect-mediated mechanism for PE-assisted transmembrane transport, sometimes referred to as the “quicksand model” [17,18]. In this model, SNAC or another PE induces transient membrane defects that become enriched in the enhancer. The polar peptide can then enter or become immersed within these PE-filled defects, which locally lowers the energetic barrier for crossing the hydrophobic membrane core. Rather than requiring formation of a stable peptide–PE complex, the model therefore proposes that transient, PE-enriched defects provide a locally favorable pathway for peptide translocation across the membrane [17,18].

### 3.3. Association with Protein Molecules

Non-covalent SNAC-peptide interactions have been confirmed through a range of biophysical methods—dynamic light scattering, surface plasmon resonance, isothermal titration calorimetry, and affinity capillary electrophoresis. It is important to note that these interactions are of low affinity: for exenatide in saline solution, the Kd is approximately 10 µM for SNAC (versus approximately 30 µM for C10), with a high contribution from non-specific interactions and rapid binding kinetics [19].

The historical hypothesis that increased lipophilicity resulting from noncovalent complexation is the primary mechanism is now a matter of debate. The more recent concept emphasizes a pH-raising, monomer-inducing, and pepsin-inhibiting effect in the stomach during oral semaglutide delivery [2,11,20].

Because SNAC is a surface-active molecule, bile salts in biorelevant intestinal media (FaSSIF and FeSSIF) weaken the interaction between SNAC and the peptide. This suggests that luminal colloids may limit the effectiveness of complexation in the small intestine [3,19,21]. Molecular dynamics simulations in the presence of taurocholate likewise show a change in the character of PE-peptide interactions. Consistent with this observation, semaglutide affects the CMC of SNAC nonmonotonically, lowering it at low concentrations and raising it at high concentrations due to oligomerization [13,19,22].

The colloidal environment of SNAC may also differ between fasted and fed intestinal conditions. In the fasted state, the lower bile salt concentration may favour greater availability of SNAC in monomeric or self-associated forms, whereas after food intake, higher bile salt concentrations promote mixed-micelle formation and alter SNAC self-association [13,21]. These effects are relevant to the intestinal fate of SNAC but should be distinguished from the principal mechanism of oral semaglutide absorption, which occurs in the stomach. The requirement for fasted administration is therefore more likely to reflect gastric factors, including the maintenance of a concentrated SNAC-semaglutide depot and the effects of food and water volume on gastric absorption [23,24]. A direct mechanistic link between intestinal SNAC-bile-salt interactions and the food effect of oral semaglutide remains speculative.

## 4. Mechanism of Action of SNAC

**Figure 2:**
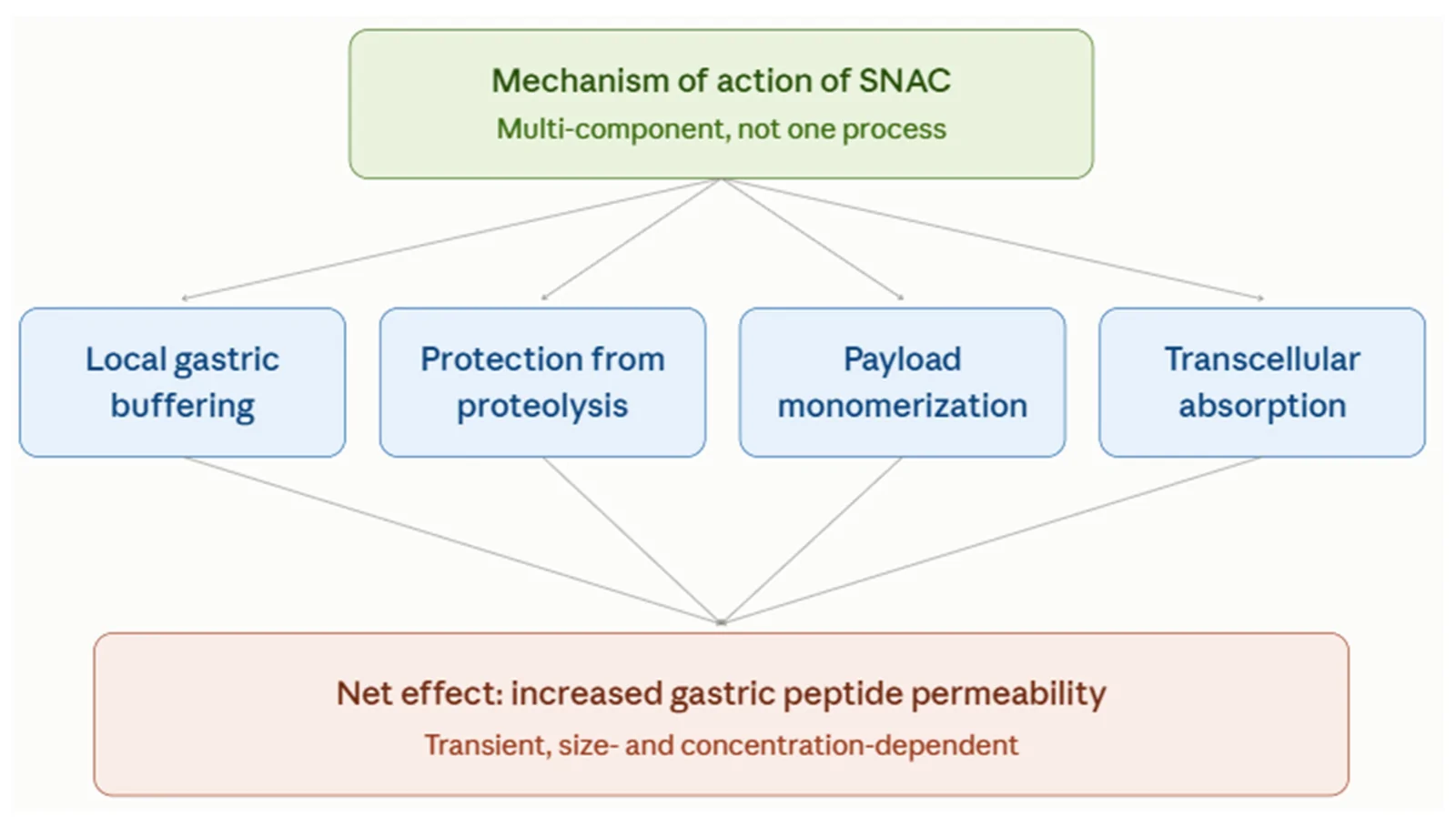
Simplified block diagram of the mechanism of action of SNAC.

The mechanism of action of SNAC is multifactorial and cannot be reduced to a single process (Figure 2). Current understanding encompasses three interrelated components: (1) local buffering of gastric contents with an increase in pH, which reduces proteolytic degradation; (2) prevention of peptide self-association and stabilization of the monomeric form; and (3) facilitation of highly localized, concentration-dependent transcellular absorption across the gastric mucosa [11,20].

### 4.1. Site of Absorption

**Figure 3:**
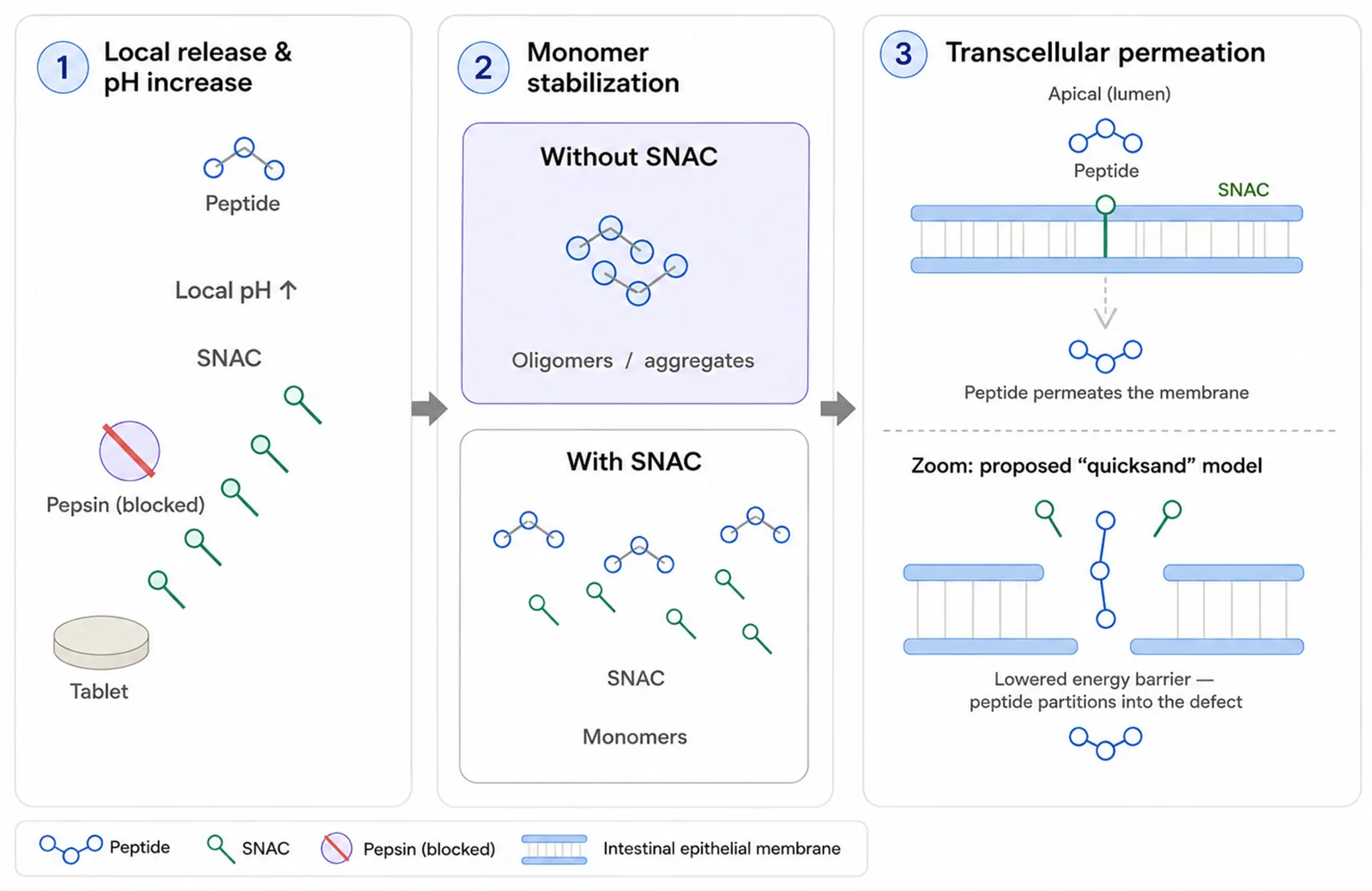
Schematic overview of the three core steps in SNAC-mediated gastric peptide absorption, illustrated using a generic peptide payload. (1) Local release and an increase in pH at the tablet–mucosa interface inactivate pepsin and protect the intact peptide. (2) In the absence of SNAC, peptide molecules self-associate into oligomers or aggregates; SNAC shifts the equilibrium toward dispersed, permeation-competent monomers. (3) SNAC interacts with the apical membrane and induces localized membrane disorder, facilitating transcellular peptide permeation. The inset illustrates the proposed “quicksand” model, in which peptide partitioning into a localized membrane defect lowers the energetic barrier to transmembrane passage. The peptide payload is depicted generically; the mechanisms shown are generalized from studies using semaglutide and other model peptides.

A defining finding for SNAC-based formulations is that absorption occurs in the stomach rather than via the conventional intestinal route characteristic of small molecules (Figure 3). In clinical and preclinical studies in dogs, semaglutide absorption is confined to a zone immediately adjacent to the tablet surface and requires co-formulation with SNAC [11]. A local interaction arises at the tablet-mucosa interface and depends on the generation of high local SNAC concentrations as the tablet erodes. This explains the need for a substantial molar excess of the enhancer relative to the peptide (300 mg SNAC per tablet of oral semaglutide) [11,25].

The localized nature of the process also explains why tablet positioning and gastric retention are critical determinants of exposure and why interindividual and intraindividual variability in absorption is high [25].

### 4.2. Local Buffering and Protection from Proteolysis

SNAC acts as a buffer, increasing the local pH in the microenvironment surrounding the tablet. This effect has a dual significance [20].

#### 4.2.1. Enzymatic Protection

Pepsin is an aspartic proteinase whose activation from pepsinogen is initiated at acidic pH through disruption of electrostatic interactions between the prosegment and the enzymatic portion [26]. Raising the pH of the gastric juice correspondingly suppresses pepsin activation. Local alkalinization by SNAC limits proteolytic degradation of the peptide payload precisely in the zone where it is released [11].

#### 4.2.2. Increased Solubility

The increased pH leads to greater peptide solubility in the microenvironment, thereby increasing the concentration gradient that drives passive permeation [25,27]. This effect is localized. SNAC does not act as an antacid on the gastric contents as a whole; rather, its clinical effect depends on maintaining a pH gradient in the immediate vicinity of the eroding tablet [11].

### 4.3. Monomerization of the Peptide Payload

The third component involves the indirect prevention of peptide self-association. Acylated GLP-1 analogs, such as semaglutide, have a pronounced tendency to oligomerize via their fatty acid side chain, and the resulting oligomers are too bulky for transcellular permeation [20]. SNAC shifts the equilibrium toward the monomeric form, which is competent for permeation [20,28]. This effect is closely linked to the amphiphilic behavior of SNAC and to the reciprocal influence of the peptide on its micellization, as described in Section 3.

### 4.4. Kinetic Characteristics: Transience, Reversibility, and Dependencies

A key aspect of the safety profile of SNAC is that it enhances absorption only transiently. The effect is time-, size-, and concentration-dependent and fully reversible. SNAC is rapidly absorbed, with a Tmax of 0.5–1 h, and has a short half-life of approximately 2 h. No accumulation is observed after repeated dosing [29].

The size dependence indicates that barrier widening is selective and does not result in indiscriminate absorption of macromolecules or microorganisms. To date, no evidence of microbial co-absorption with SNAC or C10 has been reported in clinical studies. The transient nature of the effect explains why the enhancer does not cause lasting disruption of epithelial integrity, despite the detergent-like effects observed in vitro [2,11].

Specificity is also compound-dependent. The mechanism demonstrated for semaglutide cannot necessarily be extrapolated to other peptides. The absence of an effect on tight junctions in the gastric epithelium has been confirmed in these in vivo models [11].

### 4.5. Interaction with the Mucus Layer

The mucus barrier represents a distinct and often underappreciated component of the mechanism of action of PEs. Upon exposure of ex vivo porcine intestinal mucus to SNAC, retention strength, elastic modulus, and viscosity increase, nanoparticle confinement is enhanced, and permeation of cyclosporine A and ovalbumin is reduced [30]. In contrast, the viscosity of gastric mucus and permeation through it remain unchanged in the presence of SNAC [30]. This difference is mechanistically consistent with the gastric route of absorption: SNAC does not create an additional mucus barrier precisely in the anatomical region where absorption occurs, whereas in the small intestine it may even reinforce it [11,30].

Taken together, the components described above also influence the key determinants of exposure. Semi-mechanistic pharmacokinetic modeling identifies dose, SNAC quantity, gastrointestinal permeability, and the intestinal first-pass effect as the principal factors determining the pharmacokinetics of oral semaglutide [31].

### 4.6. Other Proposed Mechanisms

Alongside the core components described above, several additional, less well-validated mechanisms have been proposed in the literature.

#### 4.6.1. Lymphatic Transport

In rats with cannulated mesenteric lymphatic vessels, Chiang et al. (2022) [32] found that co-dosing with SNAC increased the lymphatic absorption of cyanocobalamin, used as a model compound, while simultaneously increasing the concentration of SNAC itself in the lymph and systemic circulation. Subsequent studies by the same group describe a concentration-dependent effect, with a critical SNAC concentration and a minimum molar enhancer-to-payload ratio required for permeation enhancement [32]. These data pertain to an intestinal (mesenteric) route in rodents and to a small molecule. Therefore, their translatability to peptide payloads such as semaglutide, whose absorption occurs predominantly in the stomach, remains unconfirmed.

#### 4.6.2. Transcellular Transport of Non-Covalent Complexes

In early mechanistic studies within the Eligen technology, Malkov et al. (2005) showed that SNAC increases the permeability of insulin across Caco-2 monolayers approximately 10-fold, without a concomitant increase in mannitol permeability and without disruption of cell membranes [33]. Confocal microscopy and immunocytochemistry document transcellular transport without detectable changes in tight junctions, with some protection of insulin from proteolytic degradation also observed [33]. These data support a transcellular rather than a paracellular route but do not establish the involvement of a vesicular step. The subsequent hypothesis of internalization followed by accelerated basolateral exocytosis should currently be regarded as unconfirmed. Similarly, for other oral insulin delivery systems based on non-covalent complexation, the authors explicitly find no evidence of an endocytic mechanism and describe passive diffusion instead.

Overall, the strongest evidence for the mechanism of SNAC in oral semaglutide supports a localized gastric effect involving pH elevation, reduced peptide self-association, and concentration-dependent transcellular absorption. Membrane interactions are supported by experimental and biophysical studies, although the precise molecular basis of peptide passage remains incompletely resolved. By contrast, non-covalent SNAC-peptide complexation as the primary explanation for increased lipophilicity and absorption is less well supported, while the contribution of tight-junction effects appears to be context-dependent: a lack of significant tight-junction opening has been reported for semaglutide [11], whereas a paracellular component has been observed in other peptide models [34].

## 5. Clinical Data on SNAC-Based Drug Products

Clinical experience with SNAC spans a diverse set of payloads—from approved products such as oral semaglutide and cyanocobalamin, to candidates at various stages of clinical development (Table 1).

### 5.1. Oral Semaglutide

The Rybelsus R1 formulation is the first approved once-daily oral GLP-1 receptor agonist and, to date, the most extensively studied product built on SNAC technology. The tablets are oval-shaped and contain 3, 7, or 14 mg of semaglutide, co-formulated with 300 mg SNAC and the excipients microcrystalline cellulose, povidone, and magnesium stearate [25]. Absorption occurs predominantly in the stomach, and absolute bioavailability is only 0.4–1%. It is taken in a fasted state with ≤120 mL of water, at least 30 min before food or other medications. It is precisely the long half-life of semaglutide that smooths out the considerable day-to-day variability in absorption and allows clinical efficacy despite the low and variable bioavailability. This is a key consideration limiting the transferability of the platform to peptides with a short half-life [17,24,31].

Clinical efficacy has been confirmed in the PIONEER program, covering the full spectrum of T2DM [35,36]. The SOUL trial (n = 9650) showed a reduction in 3P-MACE (HR ≈ 0.86; 12.0% versus 13.8%) [37]. In the PIONEER PLUS trial, the 25 mg and 50 mg doses were superior to the 14 mg dose in terms of HbA1c reduction and body weight reduction [58]. This motivated the development of high-dose oral products containing semaglutide, while the question of achieving comparable exposure at a lower dose was addressed separately through excipient optimization in the R2 formulation (Table 2).

The R2 formulation contains 1.5, 4, or 9 mg of oral semaglutide and achieves an absolute bioavailability of 1–2% [38]. Comparable exposure equivalence has been established for the following dose pairs: 3 mg and 1.5 mg, 7 mg and 4 mg, and 14 mg and 9 mg [38]. The products are not bioequivalent on a milligram-for-milligram basis and cannot be substituted at equivalent doses. Switching between formulations is not permitted during the initiation phase (days 1–30) [38]. After this phase, switching is carried out according to the specified dose pairs, with the new formulation initiated on the following day. In the US, this formulation is marketed as Ozempic oral semaglutide tablets, while Rybelsus remains available in both formulations [38].

Mechanistically, it is significant that semaglutide absorption occurs within a narrow region of the gastric mucosa, immediately adjacent to the surface of the eroding tablet, where a transient depot with high local concentrations of SNAC and peptide is formed [11]. Accordingly, changes in tablet mass, composition, or geometry can affect erosion kinetics and the local concentration within this depot, and thereby influence bioavailability [39]. Indeed, the R2 formulation differs substantially in composition. Whereas R1 contains magnesium stearate, microcrystalline cellulose, povidone, and SNAC, R2 contains only magnesium stearate and SNAC [38]. The simplified excipient profile may alter tablet erosion and, consequently, the local concentration of SNAC at the mucosal interface. However, this mechanism remains hypothetical, as direct comparative erosion-kinetics data are not available. The difference in dose required to achieve comparable exposure (e.g., 14 mg for R1 versus 9 mg for R2, corresponding to an approximately 1.6-fold lower dose) is consistent with the higher bioavailability observed with R2, but does not by itself establish a causal relationship with tablet erosion [38].

An important consideration when comparing the different oral semaglutide formulations is the relationship between semaglutide dose, the amount of SNAC, and systemic exposure. All approved R1 strengths contain a fixed 300 mg of SNAC, despite the progressive increase in semaglutide dose from 3 to 7 to 14 mg [11]. Because the amount of SNAC is held constant while the peptide dose increases, the molar SNAC: semaglutide ratio necessarily decreases with increasing semaglutide dose. The magnitude of this decrease follows directly from the dose ratio. Increasing the semaglutide dose from 3 to 14 mg raises the peptide payload approximately 4.7-fold, so the molar ratio decreases by approximately the same factor. Using the molar masses of the sodium salt of SNAC (301.31 g/mol) and semaglutide (4113.58 g/mol) [11,25], the fixed 300 mg SNAC dose corresponds to approximately 0.996 mmol. This gives a molar SNAC: semaglutide ratio of approximately 1370:1 at 3 mg and approximately 294:1 at 14 mg. Use of the free-acid molecular mass of SNAC (~279 g/mol) would give slightly different absolute ratios. Within the fixed-SNAC R1 formulation, the SNAC:semaglutide ratio therefore decreases as the semaglutide dose increases. This changing SNAC: semaglutide ratio provides a useful framework for interpreting the dose-exposure relationship. Semaglutide exposure increases with increasing oral dose, but this should not be interpreted simply as a consequence of a proportional increase in the amount of absorption enhancer, since the enhancer dose is fixed. In early clinical pharmacology studies using a fixed 300 mg SNAC dose, single-dose exposure was maximized at the 300 mg SNAC level. At steady state, exposure was approximately twofold higher with 40 mg than with 20 mg of semaglutide, consistent with approximately dose-proportional exposure over that range [59]. Across the approved 3, 7, and 14 mg strengths, the exposure increase is close to, and at the lower end slightly greater than, proportional. The relationship is therefore best described as approximately dose-proportional rather than strictly linear. The fixed-SNAC formulation therefore permits increasing semaglutide exposure despite a progressively lower SNAC: peptide molar ratio.

If the erosion-kinetics hypothesis noted above is correct, differences in tablet composition and erosion behaviour could contribute to differences in local SNAC concentration and, consequently, absorption efficiency. Existing mechanistic modelling attributes semaglutide exposure to dose, SNAC quantity, gastrointestinal permeability, and intestinal first-pass metabolism rather than to erosion kinetics specifically [31].

This behaviour may also be related to the non-monotonic effect of semaglutide concentration on the critical micelle concentration (CMC) of SNAC. Increasing peptide concentration can lower the CMC at low concentrations but raise it at higher concentrations through peptide oligomerisation, thereby altering SNAC self-association and SNAC-peptide interactions [13]. These findings suggest that the physicochemical environment of the enhancer may vary across formulations. The clinical dose-exposure relationship should therefore be viewed as the integrated outcome of peptide dose, SNAC: peptide stoichiometry, formulation behaviour, and the efficiency of localised gastric absorption.

Similarly, the high-dose 50 mg semaglutide formulation used in the OASIS 1 trial excludes microcrystalline cellulose and povidone K90 [40]. The removal of these excipients may represent a deliberate strategy to increase bioavailability [40]. Although 9% of participants were receiving the 25 mg dose at week 68, this reflected dose reduction due to intolerance within the 50 mg arm rather than participation in a separate randomized 25 mg trial [40]. The OASIS 4 trial of 25 mg oral semaglutide (71 weeks; n = 307; 2:1 randomization versus placebo) reported a mean change in body weight at week 64 of −13.6% versus −2.2% with placebo, with ≥10% weight loss in 63.0% and ≥20% weight loss in 29.7% of patients [41]. Reductions were observed in waist circumference, HbA1c, fasting insulin, triglycerides, and CRP. Among participants with prediabetes, 71.1% achieved normoglycemia [41]. The most common adverse reactions were gastrointestinal in nature. Based on these results, the FDA approved once-daily oral semaglutide 25 mg (Wegovy) on 22 December 2025, for chronic weight management in adults with obesity or overweight and at least one weight-related comorbidity [42].

The approval of Wegovy 25 mg further extends the clinical application of SNAC technology beyond glycemic control to weight management.

### 5.2. Cyanocobalamin and SNAC

The first clinical study of B12/SNAC (Castelli et al., 2011) [43] was an open-label, randomized, parallel-group, single-dose study in 20 healthy men. Participants were divided into four groups. The four groups received 2 × 5 mg of cyanocobalamin + 100 mg of SNAC (n = 4), 1 × 5 mg of cyanocobalamin + 100 mg of SNAC (n = 6), a commercial 5 mg cyanocobalamin tablet (n = 6), or 1 mg of intravenous cyanocobalamin (n = 4), which served as the reference for absolute bioavailability. The combination with SNAC achieved an absolute bioavailability of 5.09% versus 2.16% for the commercial tablet, with markedly accelerated absorption (Tmax 0.5 h versus 6.83 h) at a practically identical elimination constant (Ke) [43]. The comparable Ke values indicate that SNAC alters the rate and extent of absorption rather than the elimination of the administered compound. The formulation was well tolerated, with no adverse reactions reported [43].

Cyanocobalamin has become established as a standard model compound for characterizing SNAC. In rats, SNAC shows a stronger effect on cyanocobalamin absorption compared with alternative enhancers (4-CNAB, 5-CNAC) [45]. SNAC itself is orally bioavailable in rats (nearly 40%) [45]. However, a directly comparable estimate of the absolute oral bioavailability of SNAC in humans has not been established in the published literature [25,35].

Co-dosing does not increase the brain penetration of quinidine or gabapentin, indicating that SNAC acts locally at the gastrointestinal level rather than systemically [45]. Studies employing mesenteric lymphatic duct cannulation further show that SNAC also enhances the lymphatic absorption of cyanocobalamin [32]. Chiang et al. (2026) determined the critical SNAC concentration and the minimal molar SNAC: cyanocobalamin ratio required for permeation enhancement [44], supporting the threshold- and ratio-dependent nature of the effect.

B12/SNAC occupies a key position in the precedent chain classically used to support the safety of SNAC in later developments, including oral semaglutide. The molecule has previously been co-formulated with heparin and vitamin B12, and is available in a marketed product (Eligen B12) [2]. The classically described mechanism involves non-covalent complexation with the drug molecule and predominantly transcellular absorption, without disruption of tight junctions or histological damage to the intestinal epithelium [46]. It should be noted that the mechanism remains debated: head-to-head comparative cell-based data characterize SNAC primarily as a surfactant with membrane effects at the high concentrations used in vivo [2].

The evidence base remains limited to a small pharmacokinetic study in healthy men, with entirely pharmacokinetic endpoints and no assessment of serum B12 as a therapeutic outcome, methylmalonic acid, homocysteine, or hematological or neurological parameters [43]. Data on clinical outcomes in patients with B12 deficiency or malabsorption are lacking. Broader reviews note that, for most B12 absorption-enhancing technologies, the evidence remains scarce [60], and that, for tablet formulations containing PEs, increases in human oral bioavailability are in the single-digit range and highly variable, with the long-term effects of repeated dosing beyond six months not having been evaluated [2].

### 5.3. Oral Unfractionated Heparin

Unfractionated heparin (UFH) is a highly sulfated, polyanionic glycosaminoglycan of high molecular mass with practically zero oral bioavailability, which has traditionally limited its use to parenteral administration [47].

Studies in Caco-2 monolayers show that SNAC enables transcellular transport of heparin. Fluorescently labeled heparin is detected intracellularly only in the presence of SNAC, without disruption of plasma membrane integrity, F-actin redistribution, or tight-junction disruption [7]. Important confirmation of the clinical relevance of this mechanism comes from analysis of molecular integrity: the disaccharide composition and molecular weight characteristics of the absorbed heparin are identical to those of parenterally administered UFH, and the anti-Xa/anti-IIa ratio remains preserved. These findings indicate that the intact, rather than depolymerized, molecule crosses the intestinal barrier [48].

In healthy volunteers, oral heparin 20,000 IU with SNAC (10.5 g) produces dose-dependent increases in aPTT (28→42.2 s), anti-Xa and anti-IIa activity, and total TFPI (from 74.9 ± 7.6 to 254.2 ± 12.3), whereas neither heparin nor SNAC alone alters hemostatic parameters. This is direct evidence that the effect is entirely enhancer-dependent [49]. Pharmacokinetically, anti-Xa activity peaks at 45–60 min and returns to baseline by 4 h, necessitating multiple daily dosing [49]. The maximum tolerated dose is limited by gastrointestinal adverse events, particularly nausea, which prompted the development of a taste-masked formulation containing 2.25 g of SNAC combined with 30,000–150,000 IU of heparin and showing good tolerability. The dose-dependent rise in aPTT and anti-Xa activity was also preserved [49]. In a preclinical rat model of deep vein thrombosis, the oral heparin/SNAC combination reduced residual DVT to 10% (versus 100% in controls), with efficacy comparable to enoxaparin [49].

In a Phase 2 trial involving 123 patients following elective total hip arthroplasty, oral heparin/SNAC (60,000 or 90,000 IU every 8 h) showed rates of venous thromboembolic events, major bleeding, and transfusion requirements similar to those observed with standard subcutaneous UFH. This is the first demonstration of safe oral heparin delivery in a postoperative setting [51].

Despite the positive results, the development did not progress to registration. The limitations are indicative of the boundaries of the platform: a disproportionately higher dose of heparin itself is required relative to the parenteral route, owing to the low fractional absorption, accompanied by gram-scale quantities of SNAC, fasting administration, and gastrointestinal adverse effects at high enhancer doses [51].

This example illustrates the core limitation of the SNAC platform: disproportionately large quantities of permeation enhancer are required for bulkier or less potent macromolecules, in contrast to compact, high-potency peptides.

### 5.4. Ibandronate and SNAC

Ibandronate is a nitrogen-containing bisphosphonate, and its oral form is approved for the treatment and prevention of postmenopausal osteoporosis [52]. Its oral bioavailability is low, at approximately 0.6%, highly variable, and markedly reduced when taken together with food [52]. For this reason, only a small fraction of the monthly 150 mg dose reaches the systemic circulation. The low absorption is attributable to the polarity of the molecule: it is negatively charged at physiological pH, which impedes transcellular transport and leaves the paracellular route as the likely predominant mechanism of absorption. This is also the basis for the dosing regimen: on an empty stomach, with water only, and no food intake for at least 60 min after the dose.

The study by McIntyre et al. (2011) was a crossover study involving 52 postmenopausal women that compared a co-formulation (ibandronate + SNAC as coated beadlets) with standalone administration and co-dosing (concurrent administration in separate formulations or SNAC administered 15 or 30 min before ibandronate) [53]. Only the co-formulation showed a 17-fold increase in Cmax and an 11-fold increase in AUC0-last relative to ibandronate alone, with accelerated absorption (tmax, 0.37 versus 0.84 h) [53]. All non-co-formulated regimens showed values practically identical to control. This demonstrates that the effect of SNAC as a PE requires genuine co-formulation, rather than mere concomitant administration.

Mechanistically, the working hypothesis is extrapolated from heparin and involves the formation of non-covalent, more lipophilic complexes that cross transcellularly without disrupting tight junctions [53]. Tolerability was good, with a similar incidence of adverse events across arms, predominantly gastrointestinal in nature (dyspepsia) and headache. All adverse events were mild, with no severe or serious adverse events, no deaths, and no discontinuations [53].

The practical implication is directly related to safety. Elderly patients with osteoporosis frequently take concomitant medications, and inadvertent simultaneous intake of another oral drug alongside the co-formulation is a realistic scenario. The results indicate that the likelihood of SNAC enhancing the absorption of a non-co-formulated concomitant substance with similar physicochemical properties is low. The authors, however, explicitly limit this conclusion to the level of gastrointestinal absorption [53].

Substantial limitations remain: the study was single-dose and open-label and had entirely pharmacokinetic endpoints. Data on bone mineral density, markers of bone turnover, or fracture outcomes are lacking, as are data on repeated dosing, and no Phase II/III trials have been conducted in patients with osteoporosis. The administered dose of ibandronate (20 mg) is far below the approved monthly dose of 150 mg, and variability remains high. The ibandronate/SNAC co-formulation has not reached the market; nevertheless, ibandronate/SNAC demonstrates substantial potential for the application of SNAC as a PE to small, highly polar molecules with low absorption. It is also the source of the mechanistically important distinction between co-formulation and co-dosing.

### 5.5. NNC0385-0434—An Oral PCSK9 Inhibitor

NNC0385-0434 is an oral PCSK9 inhibitor, a peptide based on the EGF-A domain of the human LDL receptor, developed by Novo Nordisk to lower LDL cholesterol in patients with hypercholesterolemia [54]. The peptide competitively binds free PCSK9 and prevents its binding to the LDLR, preserving receptor recycling [54]. It is administered once daily and co-formulated with 500 mg of SNAC, a substantially higher dose than the 300 mg used with oral semaglutide, illustrating the need to optimize the PE dose according to the specific peptide payload [54].

In a Phase 2 trial (NCT04992065; n = 267; patients receiving maximally tolerated statin therapy), the reduction in LDL-C at week 12 relative to placebo was 32.0% (15 mg), 44.9% (40 mg), and 61.8% (100 mg). The 100 mg dose produced a reduction comparable to that achieved with evolocumab 140 mg administered subcutaneously every 2 weeks (difference, 3.4 percentage points; 95% CI, −7.8 to 14.7). The effect approached its maximum within 2 weeks, with favorable changes also observed in apoB and Lp(a) [54]. The most common treatment-related adverse reactions were gastrointestinal (26 patients across the three NNC0385 cohorts versus 1 each for placebo and evolocumab). No deaths or related serious adverse reactions were reported [54]. Development was discontinued for portfolio reasons, despite the positive results [54].

The results with the PCSK9 inhibitor NNC0385-0434 demonstrate that SNAC can effectively achieve clinically meaningful efficacy with peptides outside the GLP-1 class as well, albeit at an enhancer dose nearly double that used with semaglutide.

### 5.6. Zenagamtide

Zenagamtide (formerly amycretin, NN9487) is a unimolecular peptide agonist of the GLP-1 and amylin receptors, with additional affinity for the calcitonin receptor [55,56,61]. It is a multireceptor peptide available for both oral and injectable administration. The oral form relies on the same gastric permeation mechanism, using SNAC as the PE, and is expected to have bioavailability of a similar magnitude to that of semaglutide [43,44]. Specific data on the bioavailability of zenagamtide in humans have not yet been published.

In a Phase 2 trial in adults with T2DM (NCT06542874; 448 participants randomized to oral and subcutaneous arms), oral zenagamtide achieved a dose-dependent reduction in HbA1c at week 36: −0.9% with 6 mg (ETD versus placebo −0.5%; *p* = 0.033), −1.3% with 25 mg (ETD −0.99%; *p* = 0.0001), and −1.4% with 50 mg (ETD −1.09%; *p* < 0.0001) [56,57]. The subcutaneous arm achieved a −1.7% reduction with 40 mg weekly (ETD, −1.56%; *p* < 0.0001), indicating greater glycemic efficacy. Serious adverse events occurred in 8% of participants in the subcutaneous arm and 4% in the oral arm [56,57].

These promising results represent the first clinical test of whether the SNAC platform can carry multi-agonist peptides at therapeutically meaningful exposures.

## 6. New Directions Beyond Metabolic Diseases

Beyond metabolic diseases, SNAC is being investigated in a number of early-stage applications that extend its use beyond classical peptide payloads and conventional solid tablet dosage forms (Table 3).

### 6.1. Chondroitin Sulfate and SNAC in a Parkinson’s Disease Model

Glycosaminoglycans possess neuroprotective potential, but their clinical application is limited by low oral bioavailability and insufficient target specificity [62]. Co-administration with SNAC significantly increases the oral bioavailability of chondroitin sulfate in animal models, with the effect surpassing that of deoxycholic acid modification [62]. Guo et al. (2026) found that in an MPTP-induced mouse model, a structurally defined 6-O-desulfated octasaccharide (CS-dp8-de6S), administered with SNAC, improved motor performance, partially restored striatal dopamine levels, and increased tyrosine hydroxylase expression in the substantia nigra, with an effect superior to that of the non-desulfated analog [62]. Mechanistically, reduced pathological deposition of fibrinogen β-chain in brain tissue was demonstrated. This represents the first evidence that SNAC can carry a payload with a neurological target [62].

### 6.2. 3D-Printed Mucoadhesive Dosage Forms with Enoxaparin

Using semisolid extrusion, dosage forms composed of Carbopol and Eudragit, containing both enoxaparin and SNAC in a single-step process without subsequent processing, have been produced. The stability of enoxaparin within the dosage form was confirmed by NMR and circular dichroism, and in vitro release was extended [63]. Ex vivo experiments demonstrate mucoadhesive properties and increased permeability across intestinal mucosa, while on a Caco-2 monolayer, the encapsulated SNAC reversibly increases transcellular transport, confirmed by transepithelial resistance measurements [63]. The approach is conceptually significant because it combines permeation enhancement with sustained local contact between the payload and enhancer, thereby addressing a limitation that is critical to the effectiveness of SNAC [63].

### 6.3. Liquid Formulations: Choline Salcaprozate (CHONAC)

An ionic liquid based on salcaprozate, termed choline salcaprozate (CHONAC), has been developed for gastric delivery of a GLP-1 analog [64]. The formulation allows for a higher quantity of PE within the same volume and faster release of both the active substance and the enhancer compared with a reference tablet, with demonstrated stability for up to 3 weeks at 4 °C [64]. In vivo, in rats and dogs, absorption is faster than with reference formulations. In dogs, however, total exposure is lower than with the tablet reference, an effect attributed to gastric physiology, likely due to dilution of the formulation and rapid transit to the duodenum, where the peptide undergoes proteolytic degradation. Repeated gastric dosing sustains absorption at a constant rate over the dosing period [64]. The authors’ conclusion is that the advantages of the liquid formulation are realized only when sustained contact between the peptide and the enhancer with the gastric epithelium is ensured [64].

Separately, engineered choline-salcaprozate ionic liquids have been investigated for non-invasive delivery of insulin and semaglutide via the intranasal and sublingual routes, achieving a hypoglycemic effect comparable to subcutaneous administration, with the enhancement mechanistically attributed to transient modulation of intercellular junctions [65].

What these three directions have in common is that they test the boundaries of the established SNAC paradigm. The spectrum of payloads is expanding (glycosaminoglycans, low-molecular-weight heparin), as are the dosage forms (3D-printed mucoadhesive matrices, ionic liquids) and routes of administration (gastric, intranasal, sublingual). Most of these data remain at the preclinical or in vitro/ex vivo stage; however, limited clinical evidence is now available for SNAC/C10 combination formulations, with human data showing substantially lower translational benefit than observed in dogs.

### 6.4. Co-Formulations of SNAC with Other Enhancers and Novel Drug Substrates

Alongside the developments described above, a substantial portion of current efforts is directed not toward new payloads, but toward combining SNAC with other PEs and toward extending its application beyond peptides.

#### 6.4.1. Combination of SNAC + Sodium Caprate (C10)

Combining the two enhancers within a single tablet has been tested for gastric delivery of a GLP-1 analog and a PCSK9 inhibitor [66]. In a cell-based gastric organoid model, the combination increases peptide permeability significantly more than either enhancer alone, and in beagle dogs, the leading SNAC/C10/diluent formulations achieve higher bioavailability than SNAC, SNAC/diluent, and C10/diluent alone [66]. In humans, however, the formulation with the PCSK9 inhibitor shows bioavailability similar to the SNAC/diluent reference. The authors attribute this result to interspecies differences in gastric pH and in the colloidal form and concentration of C10 in the gastric lumen, illustrating the poor preclinical-to-clinical translatability of C10-based systems [66].

#### 6.4.2. Erodible Tablets for Gastric Delivery of Novel Peptides

SNAC- and C10-containing erodible tablets with a dual GIP/GLP-1 agonist achieve similar absolute oral bioavailability in cynomolgus monkeys: 4.2% and 5.7%, respectively [34]. Combining a slow-release tablet design with a peptide with greater pepsin stability results in approximately fourfold higher bioavailability than that of semaglutide (4.2% versus 1.2%). Analysis of gastric tissue shows that SNAC lowers tight-junction protein levels and increases peptide uptake into the gastric epithelium, supporting a mechanism that is both paracellular and transcellular [34].

#### 6.4.3. Extension to Small Molecules of BCS Class IV

Ombitasvir is a Biopharmaceutics Classification System (BCS) class IV compound, characterized by poor solubility and low permeability. SNAC has been evaluated as an enhancer for this compound in rats and dogs, in combination with a crystalline form and amorphous solid dispersions [67]. The observed increase in bioavailability with SNAC is attributed mainly to accelerated dissolution rather than increased permeability, in contrast to lauroyl-L-carnitine and Labrasol, for which the contribution is dual [67]. This example extends the potential role of SNAC beyond macromolecular drug delivery.

#### 6.4.4. Manufacturing Aspects of Direct-Compression Dosage Forms

Comparative evaluation of C10 and SNAC as materials for direct compression shows that SNAC possesses superior compaction properties but poor flowability, whereas C10 has acceptable flowability but poor tabletability [68]. Formulations containing 72% of a single permeation enhancer (either SNAC or C10), 20% microcrystalline cellulose, 5% PVP K30, and 3% insulin fully released their contents within 30 min regardless of compression force. Upon storage at 40 °C/75% RH for one month, these formulations also showed a significant reduction in insulin deamidation and aggregation relative to the raw peptide [68].

Taken together, these developments outline three directions in the evolution of SNAC-based dosage forms: (1) synergistic enhancer combinations with, as yet, unconvincing clinical translation; (2) application to non-protein substrates; and (3) rationalization of the enhancer:drug ratio and of the technological properties of powder blends.

## 7. Discussion

The reviewed data establish SNAC as one of the most extensively clinically studied PEs, while also demonstrating clear limits to the achievable effect [2]. Clinical experience spans a diverse range of payloads, including unfractionated heparin, cyanocobalamin (a marketed product), and ibandronate (pharmacokinetic studies). Oral semaglutide is the only case to have reached broad registration and confirmed clinical outcomes [2]. Earlier-stage developments extend the spectrum to glycosaminoglycans, low-molecular-weight heparin in 3D-printed mucoadhesive matrices, and liquid ionic formulations, but all of these remain at the preclinical stage [63,64,65]. Three conclusions recur consistently across the different payloads. First, absolute bioavailability remains low and highly variable—approximately 1% for semaglutide—with pronounced interindividual and day-to-day variability [16]. For preparations containing both SNAC and sodium caprate, human studies show single-digit increases in bioavailability, which may be sufficient only for highly potent macromolecules [2]. Second, for the clinically relevant gastric formulations discussed here, the enhancing effect appears to depend strongly on co-formulation rather than simple concomitant administration; this is most clearly demonstrated with ibandronate [53]. Third, the dosing regimen is an integral part of the technology: administration in a fasted state, with a limited volume of water, and with a waiting period before food and other medications. The success of oral semaglutide highlights the decisive determinants—namely, the therapeutic potency of the payload and its tolerance for variable exposure.

### 7.1. Safety of SNAC

The safety profile of SNAC is favorable, but with clearly identified gaps. SNAC has GRAS status and has been used in the medical food Eligen Vitamin B12 [2,12,62]. In clinical studies of oral semaglutide, adverse events are predominantly gastrointestinal and are generally consistent with the known pharmacological effects of GLP-1 receptor agonism [37,41]. A specific clinical safety signal attributable to SNAC has not been established. With ibandronate, the incidence of adverse events was similar between the co-formulated and non-co-formulated arms, predominantly dyspepsia and headache, all mild in severity [52,53].

Mechanistically, the low toxicity observed in these models is consistent with the absence of substantial tight-junction disruption at concentrations relevant to permeation enhancement. In a comparative Caco-2 analysis, SNAC did not alter transepithelial resistance or increase paracellular flux except at cytotoxic concentrations, and among tight-junction-associated proteins it affects only the localization of claudin-5 [14]. In isolated rat mucosae, both enhancers produce minimal epithelial damage at flux-increasing concentrations, with potency and toxic potential characterized as low, consistent with the clinical data [15].

Preclinical toxicology outlines the dose boundaries. In a 13-week subchronic oral study in rats, mortality was observed only at 2000 mg/kg/day (20% in males and 50% in females), while in Wistar rats the NOAEL was established at 1000 mg/kg/day [9]. Slightly increased liver and kidney weights without a histopathological correlate were observed, interpreted as an adaptive response, along with gastric histopathological changes, likely secondary to irritation from the method of administration [9].

#### Impact on the Gut Microbiota

A recent preclinical study raises a novel and, to date, underexplored safety consideration for PEs. In healthy Sprague-Dawley rats treated for 21 days with semaglutide (SEM) (0.74 mg/kg/day), SNAC (22 mg/kg/day), or a SEM + SNAC combination (1:33 *w*/*w*), α-diversity remained stable, but SNAC significantly altered β-diversity (PERMANOVA, *p* < 0.05) and depleted the principal fermenting taxa *Muribaculaceae* (−62%) and *Bacteroidaceae* (−77%) relative to controls [69]. These compositional changes are associated with a reduced predicted abundance of saccharolytic enzymes and sharply reduced fecal butyrate levels (−77% with SNAC and −75% with SEM + SNAC) [69].

These changes are not confined to the intestinal lumen. Plasma analysis shows increased TNF-α (by 70%) and suppressed BDNF (by 85%) with SNAC monotherapy, and treated animals demonstrate increased liver weight and reduced cecal mass [69]. Spearman correlations link the loss of *Muribaculaceae* and *Bacteroidaceae* to reduced saccharolytic activity, lower short-chain fatty acid levels, and increased TNF-α [69]. The authors explicitly characterize the findings as associative and requiring mechanistic validation, but conclude that chronic SNAC exposure is associated with concurrent microbial, metabolic, and inflammatory changes [69].

These results carry two implications. First, they offer an additional explanation for the gastrointestinal tolerability of oral semaglutide, distinct from the purely pharmacological effect of GLP-1 agonism. Second, insofar as the data were obtained in healthy rodents, at doses and durations not directly translatable to human exposure, and without confirmation in humans, they do not warrant a change in clinical practice but do establish a priority for future research. Importantly, no corresponding clinically evident systemic inflammatory safety signal has emerged during the clinical development and use of oral semaglutide. However, the absence of such a signal should not be interpreted as evidence that SNAC has no effect on the human gut microbiome. Microbiome composition, fecal short-chain fatty acids, BDNF, and detailed inflammatory profiles are not standard endpoints in clinical trials of oral semaglutide. The available clinical evidence is therefore more accurately described as showing no clinically evident signal of harm, rather than demonstrating the absence of microbiome-related effects. Despite the accumulated clinical experience, three gaps in the safety profile of PEs remain unresolved.

The first concerns the effects of repeated exposure on the gut microbiome, which have not been systematically evaluated in humans for SNAC or sodium caprate [2]. The new preclinical microbiota data give this gap concrete substance: in healthy Sprague-Dawley rats, 21-day SNAC exposure is associated with a shift in β-diversity, depletion of the principal fermenting taxa, and a sharp reduction in fecal butyrate, accompanied by elevated plasma TNF-α and suppressed BDNF [69]. These findings offer directly testable endpoints that future long-term clinical studies should include—microbiota composition, fecal short-chain fatty acids, and levels of systemic inflammatory markers [69].

The second is the potential co-absorption of microorganisms or undesired macromolecules through the transiently increased permeability. Clinical studies to date have found no evidence of this for either SNAC or sodium caprate. For caprate, a mechanistic argument against this risk also exists: in isolated rat ileal mucosa, it does not facilitate permeation of [2] *S. typhimurium*, but rather inhibits its adhesion and invasion and exhibits bactericidal activity at millimolar concentrations [70]. The observed increase in TNF-α in the preclinical model warrants attention in this context, but is associated with the loss of primary fermenters and the decline in short-chain fatty acids, rather than with documented bacterial translocation—markers of endotoxemia (lipopolysaccharide, lipopolysaccharide-binding protein) were not measured [69]. The absence of data in this regard is not equivalent to demonstrated safety, but neither does it support a causal conclusion that translocation occurs.

The third is the potential for drug interactions. Data with ibandronate show low risk for non-co-formulated concomitant medications with similar physicochemical properties, but this conclusion is limited to the level of gastrointestinal absorption [52,53]. Beyond this, at least two additional questions remain unresolved. The first is a formulation-level interaction: aspirin, metformin, nimesulide, ciprofloxacin, and semaglutide itself significantly alter the critical micelle concentration of SNAC at intestinal pH 6.8 (baseline value 6.26 ± 0.38 mM, reduced to 3.36 mM in the presence of electrolytes) [13]. On the other hand, in direct investigation, SNAC alone does not alter the exposure of lisinopril, warfarin, digoxin, or metformin, although oral semaglutide increases the AUC of metformin without a clinically meaningful change in Cmax [71]. The second question concerns interactions at the level of metabolism and membrane transporters, for which only preclinical data are currently available. The enhancing effect of SNAC is local; the absence of change in the central exposure of model substrates argues against clinically relevant permeabilization of barriers beyond the gastrointestinal tract [44].

### 7.2. Comparison with Other Permeation Enhancers

The main comparable agent is sodium caprate (C10). The two differ primarily in mechanism, while direct evidence comparing their clinical efficacy remains limited. Sodium caprate acts through membrane perturbation and can also affect tight-junction organization. For SNAC, its effects are not limited to changes in permeability but also include modification of conditions within the gastric lumen (see Section 3). Although both SNAC and C10 share detergent-like, membrane-fluidising surfactant properties, their effects on epithelial permeability are mechanistically distinct. C10 has been shown to affect tight-junction organization and increase paracellular flux, whereas SNAC acts predominantly through membrane-mediated mechanisms [14,15]. In these ex vivo models, SNAC is less potent than C10—in isolated jejunal and colonic mucosae, 20 mM SNAC is required to achieve the efficacy of 10 mM C10. Conversely, SNAC increases permeability across isolated gastric mucosa in a concentration-dependent manner, whereas C10 does not. Molecular dynamics simulations show that SNAC lowers the central free-energy barrier to permeation more effectively than C10, but also that the two agents affect the lateral diffusion of lipids differently.

From a regulatory standpoint, SNAC holds GRAS status, whereas C10 has a long history of human use and status as a food additive. Since there is no convincing evidence of superiority of one agent over the other in terms of efficacy, the choice is determined predominantly by formulation, manufacturing, and commercial considerations.

### 7.3. Future Perspectives and Unresolved Questions

The primary unresolved limitation of SNAC is not the absence of an enhancing effect per se, but the variability of its performance under gastrointestinal conditions. Progress is likely to come from a better understanding of the physicochemical determinants of SNAC’s action under these conditions (see Section 3). Since effective membrane fluidization is expected to depend on the availability of monomeric SNAC, these findings provide a rational basis for optimizing formulations and may account for part of the observed in vivo variability [13].

The second direction is to prolong contact between the payload, the enhancer, and the epithelium. This principle unites the mucoadhesive 3D-printed matrices and the observation that the advantages of liquid formulations (CHONAC) are realized only when sustained gastric contact is ensured [64]. In dogs, CHONAC accelerates absorption, but total exposure falls short of the tablet reference due to dilution and rapid transit of the liquid to the duodenum, while repeated gastric dosing sustains a constant rate of absorption [64]. Similarly, choline-salcaprozate-based ionic liquids administered via mucosal routes act through transient modulation of intercellular junctions and exhibit a favorable biocompatibility profile [65]. The third direction is to expand the spectrum of payloads beyond peptides to glycosaminoglycans and small, highly polar molecules with a low degree of absorption, where ibandronate has already provided quantitative proof of applicability [52,53,62].

Future research should prioritize several of these unresolved issues. Long-term human studies are needed to determine whether preclinical microbiome changes associated with repeated SNAC exposure translate into clinically relevant effects in humans. The substantial variability in oral peptide bioavailability also warrants investigation of the formulation and physiological determinants of absorption, including the enhancer-to-payload ratio, gastric residence, and luminal conditions. Because SNAC performance is strongly payload- and formulation-dependent, optimization should focus on matching enhancer concentration, dosage-form design, and administration conditions to the physicochemical properties and therapeutic potency of individual payloads. Finally, next-generation SNAC derivatives and rational enhancer combinations may improve absorption efficiency, but their potential benefit remains to be established through comparative studies and clinical validation.

## 8. Conclusions

Salcaprozate sodium was incorporated into the first approved oral GLP-1 receptor agonist and has subsequently become a key component of widely used oral semaglutide formulations. The accumulated experience outlines both the potential and the limits of the platform: the effect is real and reproducible, but with low and variable bioavailability, with performance strongly dependent on genuine co-formulation and a strict dosing regimen. The platform is now entering a new phase: expansion toward new payloads beyond peptides, in parallel with the first preclinical data on its effects on the gut microbiota. The expanding scale of use makes questions regarding formulation limitations and long-term safety increasingly pressing. The answers to these questions will determine the future place of SNAC in oral drug delivery.

## Figures and Tables

**Table 1:** Summary of clinical data for SNAC-based drug products.

Compound/Product	Indication	SNAC Dose	Development Status	Key Efficacy/PK Data	Ref.
Oral semaglutide, R1 formulation (Rybelsus)	T2DM	300 mg	FDA-approved 2019 (first oral GLP-1RA)	3/7/14 mg semaglutide tablets; absolute bioavailability 0.4–1%; PIONEER program; SOUL CVOT (n = 9650): 3P-MACE HR ≈ 0.86 (12.0% vs. 13.8%)	[10,11,25,35,36,37]
Oral semaglutide, R2 formulation (Ozempic oral tablets/Rybelsus)	T2DM	R2-based; SNAC dose not reported	FDA-approved; simplified excipient profile (Mg stearate + SNAC only)	1.5/4/9 mg semaglutide tablets; bioavailability ≈ 1–2% (~2-fold R1); dose-equivalence pairs 3 mg ↔ 1.5 mg, 7 mg ↔ 4 mg, 14 mg ↔ 9 mg;	[38,39]
Oral semaglutide 25 mg (Wegovy^®^)	Chronic weight management (obesity/overweight)	R2-based (Mg stearate + SNAC only)	FDA-approved 22 Dec 2025	OASIS 4 (n = 307, 71 weeks): weight change −13.6% vs. −2.2% placebo at week 64; ≥10% loss in 63.0%, ≥20% in 29.7%; 71.1% of prediabetic participants achieved normoglycemia	[40,41,42]
Cyanocobalamin (Eligen B12)	Vitamin B12 supplementation	100 mg	Phase 1; n = 20 healthy men	Absolute bioavailability 5.09% vs. 2.16% (commercial tablet); Tmax 0.5 h vs. 6.83 h; well tolerated, no reported adverse events	[2,32,43,44,45,46]
Unfractionated heparin (UFH)	Anticoagulation/VTE prophylaxis	2.25–10.5 g	Phase 2 (post-op, n = 123); not marketed	Dose-dependent aPTT, anti-Xa, anti-IIa rise; preserved disaccharide composition & anti-Xa/anti-IIa ratio; comparable VTE/bleeding rates to s.c. UFH after hip arthroplasty	[47,48,49,50,51]
Ibandronate	Postmenopausal osteoporosis	Co-formulated (beadlets)	Single-dose crossover PK study (n = 52); not marketed	Co-formulation only: 17-fold ↑ Cmax, 11-fold ↑ AUC0-last vs. ibandronate alone; tmax 0.37 vs. 0.84 h; co-dosing (non-co-formulated) showed no enhancement	[52,53]
NNC0385-0434 (oral PCSK9 inhibitor)	Hypercholesterolemia (on maximally tolerated statin)	500 mg	Phase 2 completed (NCT04992065); discontinued for portfolio reasons	n = 267; LDL-C reduction at wk 12: −32.0% (15 mg), −44.9% (40 mg), −61.8% (100 mg); 100 mg comparable to evolocumab 140 mg SC (diff. 3.4 pp, 95% CI −7.8 to 14.7)	[54]
Zenagamtide (amycretin, NN9487)	T2DM (GLP-1/amylin/calcitonin receptor unimolecular agonist)	Not specified	Phase 2 (NCT06542874), oral (6.25 or 50 mg) daily vs. s.c. (0.4–40 mg weekly) arms	n = 448; oral (n = 186) s.c. (n = 262): HbA1c −0.9% (6 mg), −1.3% (25 mg), −1.4% (50 mg) at wk 36 vs. placebo; s.c. 40 mg: −1.7% (ETD −1.56%); no human oral bioavailability data yet published	[55,56,57]

Abbreviations: SNAC—salcaprozate sodium; FDA—U.S. Food and Drug Administration; T2DM—type 2 diabetes mellitus; UFH, unfractionated heparin; LDL-C—low-density lipoprotein cholesterol; VTE—venous thromboembolism; HbA1c—glycated hemoglobin; ETD—estimated treatment difference; MACE—major adverse cardiovascular events. The ↑ indicates “increase”.

**Table 2:** Comparison of the R1 and R2 formulations of oral semaglutide.

Parameter	R1 Formulation (Rybelsus^®^)	R2 Formulation (Ozempic Oral Tablets/Rybelsus)	Ref.
Semaglutide doses	3 mg, 7 mg, 14 mg	1.5 mg, 4 mg, 9 mg	[38]
Relative formulation	Original formulation	Optimized formulation	[38]
Excipients	SNAC 300 mg + Microcrystalline cellulose, povidone, magnesium stearate	SNAC + Magnesium stearate only	[38]
Absolute bioavailability	≈0.4–1%	≈1–2% (approx. 2-fold higher than R1)	[38]
Dose-equivalence pairs	n/a (reference formulation)	3 mg ↔ 1.5 mg; 7 mg ↔ 4 mg; 14 mg ↔ 9 mg (not bioequivalent on a mg-for-mg basis)	[38]
Switching between formulations	n/a	Not permitted during the initiation phase (days 1–30); thereafter, switch on the following day using the specified dose-equivalence pairs	[38]
Marketed brand (US)	Rybelsus^®^	Ozempic^®^ (oral tablets); Rybelsus^®^ is also available in this formulation	[38]
Proposed mechanistic basis for higher bioavailability	Reference erosion/excipient profile	Simplified excipient profile likely accelerates/alters tablet erosion kinetics, raising local SNAC concentration at the mucosal contact point at the critical moment of exposure	[39]
High-dose weight-management derivative	Not applicable	25 mg formulation (Wegovy) also excludes microcrystalline cellulose and povidone K90, consistent with the R2 excipient strategy	[40,41,42]

Note: R1 and R2 formulations are not bioequivalent on a mg-for-mg basis; see designated dose-equivalence pairs.

**Table 3:** Summary of emerging preclinical, mechanistic, and early clinical data for SNAC across cargos and applications.

Study/Platform	Cargo/Model System	Study Type	Key Findings	Ref.
I. Peptide And Macromolecular Applications				
Chondroitin sulfate + SNAC in a Parkinson’s disease model	Structurally defined 6-O-desulfated octasaccharide (CS-dp8-de6S)	In vivo, MPTP-induced mouse model	Improved motor performance and partial restoration of striatal dopamine; superior to deoxycholic-acid-modified CS	[62]
3D-printed mucoadhesive tablets	Enoxaparin (LMWH) +SNAC in a Carbopol/Eudragit matrix	In vitro/ex vivo	Enoxaparin stability confirmed; mucoadhesion and increased intestinal permeability ex vivo; reversible increase in Caco-2 transcellular transport	[63]
CHONAC (choline salcaprozate ionic liquid)	GLP-1RA	In vivo, rats and dogs	Faster absorption but lower total exposure in dogs; repeated gastric dosing sustains absorption over time	[64]
Choline-salcaprozate ionic liquids (non-gastric routes)	Insulin and semaglutide	In vivo, intranasal/sublingual delivery	Hypoglycemic effect comparable to subcutaneous administration; enhancement attributed to transient modulation of intercellular junctions	[65]
SNAC + sodium caprate (C10) combination tablets	GLP-1RA and PCSK9 inhibitor	Gastric organoids; in vivo, beagle dogs; clinical (human) arm	The combination outperformed single enhancers preclinically, but this was not replicated in humans	[66]
SNAC- and C10-containing erodible tablets	Dual GIP/GLP-1 agonist	In vivo, cynomolgus monkeys	Bioavailability: 4.2% (SNAC) vs. 5.7% (C10); approximately 4-fold higher than semaglutide, attributable to the formulation design	[34]
II. Non-Peptide Applications				
SNAC as enhancer for a BCS class IV small molecule	Ombitasvir (crystalline and amorphous solid dispersion forms)	In vivo, rats and dogs	Bioavailability increase via dissolution, not permeability enhancement	[67]
III. Other Formulations, Manufacturing, And Safety Studies				
Direct-compression tablet formulation	Insulin (72% SNAC or 72% C10, 20% MCC, 5% PVP K30, 3% insulin)	Formulation/manufacturing study	Superior compaction of SNAC vs. C10; reduced insulin degradation on storage vs. raw peptide	[68]
Gut microbiota and systemic inflammation study	Sprague-Dawley rats; 21 days; SEM 0.74 mg/kg/day, SNAC 22 mg/kg/day, or SEM + SNAC (1:33 *w*/*w*)	Safety-related in vivo preclinical study	Altered gut microbiota composition and reduced butyrate; increased TNF-α, decreased BDNF (SNAC)	[69]

Abbreviations: SNAC—salcaprozate sodium; C10—sodium caprate; LMWH—low-molecular-weight heparin; GLP-1RA—glucagon-like peptide-1 receptor agonist; PCSK9—proprotein convertase subtilisin/kexin type 9; BCS—Biopharmaceutics Classification System; MCC—microcrystalline cellulose; PVP—polyvinylpyrrolidone; MPTP—1-methyl-4-phenyl-1,2,3,6-tetrahydropyridine; Caco-2—human colorectal adenocarcinoma cell line.

## Data Availability

No new data were created or analyzed in this study. Data sharing is not applicable to this article.

## Abbreviations

aPTT	Activated partial thromboplastin time
apoB	Apolipoprotein B
BCS	Biopharmaceutics Classification System
BDNF	Brain-derived neurotrophic factor
C10	Sodium caprate
CD	Circular dichroism
CHONAC	Choline salcaprozate (ionic liquid)
CI	Confidence interval
Cmax	Maximum plasma concentration
CMC	Critical micelle concentration
CRP	C-reactive protein
DMPC	Dimethylphosphatidylcholine
DVT	Deep vein thrombosis
EGF-A	Epidermal growth factor A (domain)
EMA	European Medicines Agency
ETD	Estimated treatment difference
FD4	FITC-dextran 4000
FDA	U.S. Food and Drug Administration
FaSSIF	Fasted State Simulated Intestinal Fluid
FeSSIF	Fed State Simulated Intestinal Fluid
FITC	Fluorescein isothiocyanate
GI	Gastrointestinal
GIP	Glucose-dependent insulinotropic polypeptide
GLP-1	Glucagon-like peptide-1
GLP-1RA(s)	GLP-1 receptor agonist(s)
GRAS	Generally recognized as safe
HbA1c	Glycated hemoglobin
HR	Hazard ratio
IU	International units
i.v.	Intravenous
Kd	Dissociation constant
LDL	Low-density lipoprotein
LDL-C	LDL cholesterol
LDLR	LDL receptor
LMWH	Low-molecular-weight heparin
Lp(a)	Lipoprotein(a)
MACE	Major adverse cardiovascular events
MCC	Microcrystalline cellulose
MPTP	1-Methyl-4-phenyl-1,2,3,6-tetrahydropyridine
NMR	Nuclear magnetic resonance
NOAEL	No observed adverse effect level
PCSK9	Proprotein convertase subtilisin/kexin type 9
PE(s)	Permeation enhancer(s)
Papp	Apparent permeability coefficient
PERMANOVA	Permutational multivariate analysis of variance
pKa	Acid dissociation constant
PVP	Polyvinylpyrrolidone (povidone)
RH	Relative humidity
s.c.	Subcutaneous
SEM	Semaglutide
ssNMR	Solid-state nuclear magnetic resonance
SNAC	Salcaprozate sodium (sodium N-[8-(2-hydroxybenzoyl)amino]caprylate)
T2DM	Type 2 diabetes mellitus
TEER	Transepithelial electrical resistance
TFPI	Tissue factor pathway inhibitor
TNF-α	Tumor necrosis factor alpha
UFH	Unfractionated heparin
VTE	Venous thromboembolism

## References

[1] Zhu Q., Chen Z., Paul P.K., Lu Y., Wu W., Qi J. (2021). Oral Delivery of Proteins and Peptides: Challenges, Status Quo and Future Perspectives. Acta Pharm. Sin. B.

[2] Twarog C., Fattah S., Heade J., Maher S., Fattal E., Brayden D.J. (2019). Intestinal Permeation Enhancers for Oral Delivery of Macromolecules: A Comparison between Salcaprozate Sodium (SNAC) and Sodium Caprate (C10). Pharmaceutics.

[3] Maher S., Mrsny R.J., Brayden D.J. (2016). Intestinal Permeation Enhancers for Oral Peptide Delivery. Adv. Drug Deliv. Rev..

[4] Asano D., Takakusa H., Nakai D. (2023). Oral Absorption of Middle-to-Large Molecules and Its Improvement, with a Focus on New Modality Drugs. Pharmaceutics.

[5] Mehrotra S., Kalyan Bg P., Nayak P.G., Joseph A., Manikkath J. (2024). Recent Progress in the Oral Delivery of Therapeutic Peptides and Proteins: Overview of Pharmaceutical Strategies to Overcome Absorption Hurdles. Adv. Pharm. Bull..

[6] Kim J.C., Park E.J., Na D.H. (2022). Gastrointestinal Permeation Enhancers for the Development of Oral Peptide Pharmaceuticals. Pharmaceuticals.

[7] Kommineni N., Sainaga Jyothi V.G.S., Butreddy A., Raju S., Shapira T., Khan W., Angsantikul P., Domb A.J. (2023). SNAC for Enhanced Oral Bioavailability: An Updated Review. Pharm. Res..

[8] Leone-Bay A., Santiago N., Achan D., Chaudhary K., DeMorin F., Falzarano L., Haas S., Kalbag S., Kaplan D. (1995). N-Acylated .Alpha.-Amino Acids as Novel Oral Delivery Agents for Proteins. J. Med. Chem..

[9] Riley M.G.I., Castelli M.C., Paehler E.A. (2009). Subchronic Oral Toxicity of Salcaprozate Sodium (SNAC) in Sprague-Dawley and Wistar Rats. Int. J. Toxicol..

[10] Lewis A.L., McEntee N., Holland J., Patel A. (2022). Development and Approval of Rybelsus (Oral Semaglutide): Ushering in a New Era in Peptide Delivery. Drug Deliv. Transl. Res..

[11] Buckley S.T., Bækdal T.A., Vegge A., Maarbjerg S.J., Pyke C., Ahnfelt-Rønne J., Madsen K.G., Schéele S.G., Alanentalo T., Kirk R.K. (2018). Transcellular Stomach Absorption of a Derivatized Glucagon-like Peptide-1 Receptor Agonist. Sci. Transl. Med..

[12] Fattah S., Ismaiel M., Murphy B., Rulikowska A., Frias J.M., Winter D.C., Brayden D.J. (2020). Salcaprozate Sodium (SNAC) Enhances Permeability of Octreotide across Isolated Rat and Human Intestinal Epithelial Mucosae in Ussing Chambers. Eur. J. Pharm. Sci..

[13] Akbar S., Joseph A., Malgave A., Kumar A., Bej P., Malayandi R. (2026). Influence of pH, Buffering Capacity, Ionic Strength, Bile Salts, and Model Drugs on Micellization Behaviour of Salcaprozate Sodium in Physiological Range pH Buffers. AAPS PharmSciTech.

[14] Twarog C., Liu K., O’Brien P.J., Dawson K.A., Fattal E., Illel B., Brayden D.J. (2020). A Head-to-Head Caco-2 Assay Comparison of the Mechanisms of Action of the Intestinal Permeation Enhancers: SNAC and Sodium Caprate (C10). Eur. J. Pharm. Biopharm..

[15] Twarog C., McCartney F., Harrison S.M., Illel B., Fattal E., Brayden D.J. (2021). Comparison of the Effects of the Intestinal Permeation Enhancers, SNAC and Sodium Caprate (C10): Isolated Rat Intestinal Mucosae and Sacs. Eur. J. Pharm. Sci..

[16] Ling J., Schroder R., Wuelfing W.P., Higgins J., Kesisoglou F., Templeton A.C., Su Y. (2025). Molecular Investigation of SNAC as an Oral Peptide Permeation Enhancer in Lipid Membranes via Solid-State NMR. Mol. Pharm..

[17] Colston K.J., Faivre K.T., Schneebeli S.T. (2025). Permeation Enhancer-Induced Membrane Defects Assist the Oral Absorption of Peptide Drugs. Nat. Commun..

[18] Bernaldez M., Kang C., Stamatis S.D., Rose J.P., Sun R. (2025). The Impact of Permeation Enhancers on Transcellular Permeation of Small Molecule Drugs. J. Phys. Chem. B.

[19] Twarog C., Fattal E., Noiray M., Illel B., Brayden D.J., Taverna M., Hillaireau H. (2022). Characterization of the Physicochemical Interactions between Exenatide and Two Intestinal Permeation Enhancers: Sodium Caprate (C10) and Salcaprozate Sodium (SNAC). Int. J. Pharm..

[20] Meier J.J., Granhall C., Hoevelmann U., Navarria A., Plum-Moerschel L., Ramesh C., Tannapfel A., Kapitza C. (2022). Effect of Upper Gastrointestinal Disease on the Pharmacokinetics of Oral Semaglutide in Subjects with Type 2 Diabetes. Diabetes Obes. Metab..

[21] Hossain S., Joyce P., Parrow A., Jõemetsa S., Höök F., Larsson P., Bergström C.A.S. (2020). Influence of Bile Composition on Membrane Incorporation of Transient Permeability Enhancers. Mol. Pharm..

[22] Hossain S., Kneiszl R., Larsson P. (2023). Revealing the Interaction between Peptide Drugs and Permeation Enhancers in the Presence of Intestinal Bile Salts. Nanoscale.

[23] Overgaard R.V., Navarria A., Ingwersen S.H., Bækdal T.A., Kildemoes R.J. (2021). Clinical Pharmacokinetics of Oral Semaglutide: Analyses of Data from Clinical Pharmacology Trials. Clin. Pharmacokinet..

[24] Van Hout M., Forte P., Jensen T.B., Boschini C., Bækdal T.A. (2023). Effect of Various Dosing Schedules on the Pharmacokinetics of Oral Semaglutide: A Randomised Trial in Healthy Subjects. Clin. Pharmacokinet..

[25] Bain E.K., Bain S.C. (2021). Recent Developments in GLP-1RA Therapy: A Review of the Latest Evidence of Efficacy and Safety and Differences within the Class. Diabetes Obes. Metab..

[26] Richter C., Tanaka T., Yada R.Y. (1998). Mechanism of Activation of the Gastric Aspartic Proteinases: Pepsinogen, Progastricsin and Prochymosin. Biochem. J..

[27] Davies M., Pieber T.R., Hartoft-Nielsen M.-L., Hansen O.K.H., Jabbour S., Rosenstock J. (2017). Effect of Oral Semaglutide Compared with Placebo and Subcutaneous Semaglutide on Glycemic Control in Patients with Type 2 Diabetes: A Randomized Clinical Trial. JAMA.

[28] Forst T., De Block C., Del Prato S., Armani S., Frias J., Lautenbach A., Ludvik B., Marinez M., Mathieu C., Müller T.D. (2024). The Role of Incretin Receptor Agonists in the Treatment of Obesity. Diabetes Obes. Metab..

[29] European Medicines Agency (2020). Rybelsus: Assessment Report.

[30] Mortensen J.S., Bohr S.S.-R., Harloff-Helleberg S., Hatzakis N.S., Saaby L., Nielsen H.M. (2022). Physical and Barrier Changes in Gastrointestinal Mucus Induced by the Permeation Enhancer Sodium 8-[(2-Hydroxybenzoyl)Amino]Octanoate (SNAC). J. Control. Release.

[31] Tiwari A., Akbar S., Joseph A., Malayandi R., Chilamakuri S.N., Natesan S. (2026). Steady-State Pharmacokinetics of Oral Semaglutide Using Semi-Mechanistic Pharmacokinetic Modelling and Population Simulations. Xenobiotica.

[32] Chiang P.-C., Liu J., Nagapudi K., Wu R., Dolton M., Chen J., Plise E., Liu L., Durk M.R. (2022). Elucidating a Potential Mechanism of Permeability Enhancer Sodium N-[8-(2-Hydroxybenzoyl) Amino] Caprylate in Rats: Evidence of Lymphatic Absorption of Cyanocobalamin Using the Mesenteric Lymph Duct Cannulated Rat. J. Pharm. Sci..

[33] Malkov D., Angelo R., Wang H., Flanders E., Tang H., Gomez-Orellana I. (2005). Oral Delivery of Insulin with the Eligen(®) Technology: Mechanistic Studies. CDD.

[34] Tran H., Dogra M., Huang S., Aihara E., ElSayed M., Aburub A. (2024). Development and Evaluation of C10 and SNAC Erodible Tablets for Gastric Delivery of a GIP/GLP1 Peptide in Monkeys. Int. J. Pharm..

[35] Thethi T.K., Pratley R., Meier J.J. (2020). Efficacy, Safety and Cardiovascular Outcomes of Once-daily Oral Semaglutide in Patients with Type 2 Diabetes: The PIONEER Programme. Diabetes Obes. Metab..

[36] Rodbard H.W., Dougherty T., Taddei-Allen P. (2020). Efficacy of Oral Semaglutide: Overview of the PIONEER Clinical Trial Program and Implications for Managed Care. Am. J. Manag. Care.

[37] McGuire D.K., Marx N., Mulvagh S.L., Deanfield J.E., Inzucchi S.E., Pop-Busui R., Mann J.F.E., Emerson S.S., Poulter N.R., Engelmann M.D.M. (2025). Oral Semaglutide and Cardiovascular Outcomes in High-Risk Type 2 Diabetes. N. Engl. J. Med..

[38] U.S. Food and Drug Administration (2026). RYBELSUS and OZEMPIC (Semaglutide) Tablets-Highlights of Prescribing Information.

[39] Kim D.-H., Hwang S.-K., Yoon J.-H., Na D.H., Park Y.-J., Chae Y.-J., Chang J.-E., Kim J.-E. (2026). Formulation Engineering of Oral Semaglutide Tablets: Unleashing Gastric Intestinal Permeation with Sodium Caprate. Pharmaceutics.

[40] Knop F.K., Aroda V.R., Do Vale R.D., Holst-Hansen T., Laursen P.N., Rosenstock J., Rubino D.M., Garvey W.T. (2023). Oral Semaglutide 50 Mg Taken Once per Day in Adults with Overweight or Obesity (OASIS 1): A Randomised, Double-Blind, Placebo-Controlled, Phase 3 Trial. Lancet.

[41] Wharton S., Lingvay I., Bogdanski P., Duque Do Vale R., Jacob S., Karlsson T., Shaji C., Rubino D., Garvey W.T. (2025). Oral Semaglutide at a Dose of 25 Mg in Adults with Overweight or Obesity. N. Engl. J. Med..

[42] U.S. Food and Drug Administration (2026). Wegovy FDA Drug Label.

[43] Castelli M.C., Wong D.F., Friedman K., Riley M.G.I. (2011). Pharmacokinetics of Oral Cyanocobalamin Formulated with Sodium N-[8-(2-Hydroxybenzoyl)Amino]Caprylate (SNAC): An Open-Label, Randomized, Single-Dose, Parallel-Group Study in Healthy Male Subjects. Clin. Ther..

[44] Chiang P.-C., Liu J., Nagapudi K., Pang J., Plise E., Dolton M., Durk M.R. (2026). Investigating the SNAC-to-Drug Ratio for the Oral Absorption of Cyanocobalamin in Rats. J. Pharm. Sci..

[45] Chiang P.-C., Deshmukh G., Liu J., Nagapudi K., Chen J.Z., Valle N., Li R., Plise E.G., Durk M.R. (2020). Evaluating the Pharmacokinetics and Systemic Effects of a Permeability Enhancer Sodium N-[8-(2-Hydroxybenzoyl)Amino] Caprylate in Rats. J. Pharm. Sci..

[46] Verma S., Goand U.K., Husain A., Katekar R.A., Garg R., Gayen J.R. (2021). Challenges of Peptide and Protein Drug Delivery by Oral Route: Current Strategies to Improve the Bioavailability. Drug Dev. Res..

[47] Pineo G., Hull R., Marder V. (2004). Oral Delivery of Heparin: SNAC and Related Formulations. Best. Pract. Res. Clin. Haematol..

[48] Mousa S.A., Zhang F., Aljada A., Chaturvedi S., Takieddin M., Zhang H., Chi L., Castelli M.C., Friedman K., Goldberg M.M. (2007). Pharmacokinetics and Pharmacodynamics of Oral Heparin Solid Dosage Form in Healthy Human Subjects. J. Clin. Pharmacol..

[49] Baughman R.A., Kapoor S.C., Agarwal R.K., Kisicki J., Catella-Lawson F., FitzGerald G.A. (1998). Oral Delivery of Anticoagulant Doses of Heparin: A Randomized, Double-Blind, Controlled Study in Humans. Circulation.

[50] Gonze M.D., Salartash K., Sternbergh W.C., Baughman R.A., Leone-Bay A., Money S.R. (2000). Orally Administered Unfractionated Heparin with Carrier Agent Is Therapeutic for Deep Venous Thrombosis. Circulation.

[51] Berkowitz S.D., Marder V.J., Kosutic G., Baughman R.A. (2003). Oral Heparin Administration with a Novel Drug Delivery Agent (SNAC) in Healthy Volunteers and Patients Undergoing Elective Total Hip Arthroplasty. J. Thromb. Haemost..

[52] Frampton J.E., Perry C.M. (2008). Ibandronate: A Review of Its Use in the Management of Postmenopausal Osteoporosis. Drugs.

[53] McIntyre C., Schmidt J., Castelli M.C., Bittner B. (2011). Study on the Impact of SNAC (Sodium N-[8-(2-Hydroxybenzoyl) Amino] Caprylate) on the Bioavailability of Ibandronate (IBN) in Postmenopausal Women. J. Drug Deliv. Sci. Technol..

[54] Koren M.J., Descamps O., Hata Y., Hengeveld E.M., Hovingh G.K., Ikonomidis I., Radu Juul Jensen M.D., Langbakke I.H., Martens F.M.A.C., Søndergaard A.L. (2024). PCSK9 Inhibition with Orally Administered NNC0385-0434 in Hypercholesterolaemia: A Randomised, Double-Blind, Placebo-Controlled and Active-Controlled Phase 2 Trial. Lancet Diabetes Endocrinol..

[55] Fu L., Ding R., Xu G., Hu J., Yang P. (2026). Amycretin in Obesity: Mechanisms, Clinical Efficacy, and Future Perspectives. Metabolism.

[56] Mora P., Aroda V.R., Asong M., Blüher M., Carlander A.-L.F., Kaltoft M., Vilsen L.N., Rosenstock J. (2026). Efficacy and Safety of Once-Daily Oral Zenagamtide, a Novel Unimolecular GLP-1 and Amylin Receptor Agonist, in Adults with Type 2 Diabetes: A Multicentre, Randomised, Parallel, Double-Blind, Placebo-Controlled, Dose-Finding, Phase 2 Trial. Lancet.

[57] Mora P., Aroda V.R., Asong M., Blüher M., Heftdal L.D., Carlander A.-L.F., Vilsen L.N., Rosenstock J. (2026). Efficacy and Safety of Once-Weekly Subcutaneous Zenagamtide, a Novel Unimolecular GLP-1 and Amylin Receptor Agonist, in Type 2 Diabetes: A Multicentre, Randomised, Parallel, Double-Blind, Placebo-Controlled, Dose-Finding, Phase 2 Trial. Lancet.

[58] Aroda V.R., Aberle J., Bardtrum L., Christiansen E., Knop F.K., Gabery S., Pedersen S.D., Buse J.B. (2023). Efficacy and Safety of Once-Daily Oral Semaglutide 25 Mg and 50 Mg Compared with 14 Mg in Adults with Type 2 Diabetes (PIONEER PLUS): A Multicentre, Randomised, Phase 3b Trial. Lancet.

[59] Granhall C., Donsmark M., Blicher T.M., Golor G., Søndergaard F.L., Thomsen M., Bækdal T.A. (2019). Safety and Pharmacokinetics of Single and Multiple Ascending Doses of the Novel Oral Human GLP-1 Analogue, Oral Semaglutide, in Healthy Subjects and Subjects with Type 2 Diabetes. Clin. Pharmacokinet..

[60] Brito A., Habeych E., Silva-Zolezzi I., Galaffu N., Allen L.H. (2018). Methods to Assess Vitamin B12 Bioavailability and Technologies to Enhance Its Absorption. Nutr. Rev..

[61] Buch A., Eldor R., Brown R., Zonszein J. (2025). Novel Oral Agents in Anti-Obesity Pharmacotherapy: A Narrative Review. Diabetes Obes. Metab..

[62] Guo Y., Zhang B., Bu C., An Z., Shi D., Jin L., Sheng A., Zhen A., Chi L. (2026). Oral Efficacy of Structurally Defined Chondroitin Sulfate Co-Administered with SNAC in Parkinson’s Disease. Carbohydr. Polym..

[63] Chatzitaki A.-T., Patila M., Haralampos S., Vizirianakis I.S., Rekka E.A., Tzetzis D., Spyros A., Zacharis C.K., Ritzoulis C., Fatouros D.G. (2024). Development of Mucoadhesive 3D-Printed Carbopol/Eudragit/SNAC Tablets for the Oral Delivery of Enoxaparin: In Vitro and Ex Vivo Evaluation. Int. J. Pharm..

[64] Rebollo R., Niu Z., Blaabjerg L., La Zara D., Juel T., Pedersen H.D., Andersson V., Benova M., Krogh C., Pons R. (2025). Salcaprozate-Based Ionic Liquids for GLP-1 Gastric Delivery: A Mechanistic Understanding of in Vivo Performance. J. Control. Release.

[65] Huang R., Yu J., Zhang B., Ran W., Zhao M., An N., Ye J., Li J., Cao E., Wang Y. (2026). Engineered Choline-Salcaprozate Ionic Liquids for Enhanced Non-Invasive Delivery of Macromolecular Antidiabetics. J. Colloid Interface Sci..

[66] Niu Z., La Zara D., Blaabjerg L., Pessi J., Raptis K., Toftlev A., Sauter M., Christophersen P., Bardonnet P.-L., Andersson V. (2025). Combining SNAC and C10 in Oral Tablet Formulations for Gastric Peptide Delivery: A Preclinical and Clinical Study. J. Control. Release.

[67] Indulkar A.S., Hanouch L., Gignac N., Borchardt T., Marsh K. (2025). Investigating the Effect of Permeation Enhancers on Oral Absorption of a BCS IV Compound, Ombitasvir, Utilizing Animal Models. J. Pharm. Sci..

[68] Fagan A., Bateman L.M., O’Mahony M., Crean A.M., O’Shea J.P. (2025). Direct Compression Peptide Tablets: Insights into the Importance of Permeation Enhancer Processibility and Peptide Stability. Int. J. Pharm..

[69] Ariaee A., Noueihad K., Hunter A., Wignall A., Wardill H.R., Davies M., Prestidge C.A., Joyce P. (2026). Gut Microbiota Perturbation and Systemic Inflammation Are Associated with Salcaprozate Sodium (SNAC)-Enabled Oral Semaglutide Delivery. J. Control. Release.

[70] Cox A.B., Rawlinson L.-A., Baird A.W., Bzik V., Brayden D.J. (2008). In Vitro Interactions Between the Oral Absorption Promoter, Sodium Caprate (C10) and S. Typhimurium in Rat Intestinal Ileal Mucosae. Pharm. Res..

[71] Bækdal T.A., Borregaard J., Hansen C.W., Thomsen M., Anderson T.W. (2019). Effect of Oral Semaglutide on the Pharmacokinetics of Lisinopril, Warfarin, Digoxin, and Metformin in Healthy Subjects. Clin. Pharmacokinet..

